# Does concomitant ductal carcinoma in situ affect the clinical outcome in breast cancer patients with invasive ductal carcinoma: An Asian perspective

**DOI:** 10.1002/cnr2.1646

**Published:** 2022-07-26

**Authors:** Wai Peng Lee, Spoorthi Sudhakar Shetty, Chin Mui Jaime Seah, Pei Ting Tan, Su Ming Tan

**Affiliations:** ^1^ Division of Breast Surgery, Department of Surgery Changi General Hospital Singapore Singapore; ^2^ Clinical Trials and Research Unit Changi General Hospital Singapore Singapore

**Keywords:** breast cancer, breast tumor, disease, ductal carcinoma in situ, invasive breast cancer

## Abstract

**Background:**

Ductal carcinoma in situ (DCIS) is an established precursor to invasive ductal carcinoma (IDC) and its coexistence with IDC appear to favor reduced biological aggressiveness. Its prognostic implication and ability to affect clinical outcome has been understudied in Asia. This study aims to explore if concomitant DCIS affects the clinical behavior and outcomes among Asians.

**Aim:**

Stages I to III breast cancer patients with histological proven IDC, diagnosed and treated in a single institution from June 1, 2004 to June 30, 2014 were included in this study. Statistical analyses were conducted using *Χ* ^2^ test, independent *t* test, multivariate logistic regression and Kaplan–Meier test.

**Methods and Results:**

A total of 818 patients were identified, including 224 and 594 patients with isolated IDC (No‐DCIS) and IDC with coexisting DCIS (IDC‐DCIS) respectively. Patients with IDC‐DCIS were found to have smaller tumors (median: 22 mm, *p* ≤ .01), estrogen receptor positivity (*p* = .001), progesterone receptor positivity (*p* < .001) and associated with better pathological stage (*p* = .001). Patients with No‐DCIS were 1.6 times more likely to develop disease progression (95% CI: 1.1–2.3, *p* = .027) and subsequently associated with distant recurrences (20.5% vs. 13.6%, *p* = .02). The breast cancer specific 5 year overall survival rate for patients with No‐DCIS and those with IDC‐DCIS was 90.9% (95% CI: 86.2%–94.5%) and 93.7% (95% CI: 91.4%–95.5%), respectively (*p* = .202).

**Conclusion:**

The presence of DCIS component in IDC among Asians is associated with favorable tumor biological profile, thereby indicating reduced disease aggressiveness. Our study is the first to report the clinical significance in terms of disease progression and distant recurrences among Asians.

## 1 INTRODUCTION

Screening mammography has led to a rising detection of early breast cancer, namely ductal carcinoma in situ (DCIS). Though it has been assumed that invasive cancers are likely to have derived from pre‐existing DCIS,^1^, ^2^ IDC may still evolve de novo in up to 21.4% of cases worldwide. A delay in transformation from in situ to invasive form is believed to account for tumors with coexisting DCIS demonstrating lesser biological aggresiveness.^3^, ^4^ Prior studies have linked tumors with IDC‐DCIS with better clinical features such as smaller and lower grade tumors and lower probability of lymph node invasion. Nonetheless, it remains controversial if clinical outcomes such as recurrences and overall survival rates are affected.^5^, ^6^ The clinical impact of concomitant DCIS in invasive cancers involving Asians has been understudied. Hence, our study aims to address if coexisting DCIS affects the tumor characteristics and clinical outcome such as recurrences, disease progression and overall survival among Asians.

## 2 MATERIALS AND METHODS

**FIGURE 1:**
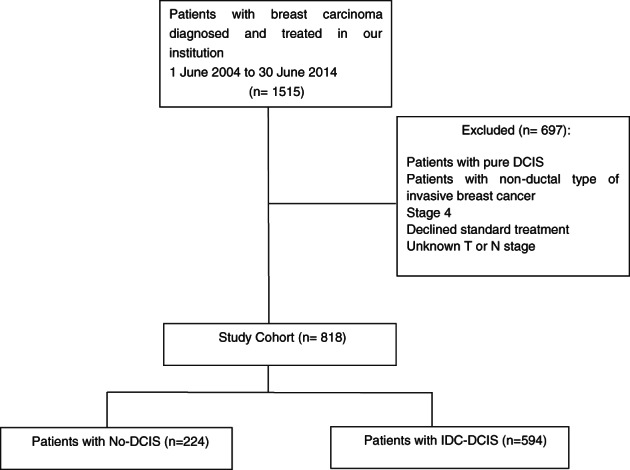
Study cohort flow diagram

A retrospective analysis was performed on our prospectively collected breast cancer database with an inclusion period from June 1, 2004 to June 30, 2014. This database comprises of patients who were diagnosed and underwent treatment in a specialized breast unit of a single hospital institution. Only patients with definitive histopathology diagnosis for invasive ductal carcinoma (IDC) were selected for evaluation. Patients diagnosed with metastatic disease were excluded. Patients with bilateral breast cancer were included as two separate study cases. Figure 1 showed the inclusion and exclusion criteria that resulted in our main study cohort of 818 patients.

Data collected include clinical characteristics, histopathological information and the type of surgery performed. The TNM classification was based on the latest edition of the American Joint Committee on Cancer at the time of reporting of the histopathology specimen.

Sentinel lymph node biopsy (SLNB) was performed in all patients with a preoperative diagnosis of infiltrative ductal carcinoma and in the absence of preoperative clinical or radiological evidence of nodal involvement. Axillary dissection was performed in patients who had preoperative diagnosis of axillary lymph node metastasis or macro metastasis on frozen section. Tumor histopathology and the number of lymph nodes involved were evaluated by routine hematoxylin‐eosin (H& E) staining.

The cases were divided into two groups: invasive ductal carcinoma (No‐DCIS) and invasive ductal carcinoma with ductal carcinoma in situ (IDC‐DCIS).

This study had received the approval of the institutional ethics committee (IRB Ref No: 2019/2884).

Quantitative data are shown as median or mean of their values and their variability is expressed as range or *SD*, as specified for each analysis. Qualitative values are shown as absolute values or percentages. Categorical data were presented in frequency and percentage and association between subjects' characteristics and DCIS were tested using chi‐square test.

Numerical data was presented in mean (standard deviation) and association was tested using independent *t* test if normal distribution was fulfilled. Otherwise, data were presented in median and inter‐quartile rage (IQR) and Mann–Whitney *U* test was performed to test for the association between subjects' characteristics and DCIS status. Logistic regression was performed to identify the risk of developing disease progression between DCIS and non‐DCIS patients.

Survival analysis and duration to disease free progression between DCIS and non‐DCIS were performed using Kaplan–Meier with log rank test to assess for statistical differences.

Propensity scores were calculated using a multiple Logistic Regression with clinical relevant variables: age, overall tumor stage, menopausal status, hormonal status, and cerB2 receptor status. Nearest‐neighbor matching with maximum difference of 5% approach in 1:1 fashion was performed to match the propensity scores values of the DCIS and non‐DCIS group.

Statistical significance was set at *p* < .05. Analysis was performed using SPSS version 21 (IBM Corp., Armonk).

## 3 RESULTS

A total of 818 cases from June 1, 2004 to June 30, 2014 were included. No‐DCIS was present in 224 (27.4%) cases while 594 (72.6%) had IDC‐DCIS. The mean age at diagnosis was 55.6 years (42.1–69.1). The mean follow up period in our study was 93.7 months (47.2–140.2). The ethnic distribution of our study cohort was consistent with our national demographics. The clinicopathological features and the analysis between the two groups are shown in Table 1. Tumors were either T1 (43.8%) or T2 (46.3%). Tumors which were T3 made up 9.8% of the study population. Five hundred and one patients (61.2%) had no nodal involvement (N0). Patients were found to have Stage 1 (32.6%) and 2 (53.2%) cancer, followed by Stage 3 (14.2%). Patients with Stage 4 disease were excluded in the analysis due to their limited prognosis.

**TABLE 1:** Clinicopathologic features of the entire study population and No‐DCIS and IDC‐DCIS study groups

Variable	Total (*n* = 818)	No‐DCIS (*n* = 224)	IDC‐DCIS (*n* = 594)	*p*‐value
**Age mean years (*SD*)**	55.6 (13.5)	55.3 (14.5)	55.7 (13.1)	*p* = .739
**Age group**				
<40	82 (10.0)	31 (14.5)	51 (8.6)	*p* = .826
40–49	224 (27.3)	50 (23.3)	174 (29.3)	
50–59	218 (26.7)	61 (28.5)	157 (26.4)	
60–69	147 (18.0)	41 (19.2)	106 (17.8)	
≥70	147 (18.0)	31 (14.5)	106 (17.8)	
**Ethnicity**				
Chinese	588 (71.9)	162 (72.3)	426 (71.7)	*p* = .397
Malay	135 (16.5)	42 (18.8)	93 (15.7)	
Indian	46 (5.6)	9 (4.0)	37 (6.2)	
Others	49 (6.0)	11 (4.9)	38 (6.4)	
**Follow‐up duration** Mean (*SD*)	93.7 (46.5)	91.6 (49.3)	93.8 (45.5)	*p* = .541
**Invasive tumor size, median (mm)**	24 (15–35)	27 (18–45)	22 (14–31.3)	***p < .001***
**Tumor status *n* (%)**				
1	358 (43.8)	78 (21.8)	280 (78.2)	***p < .001***
2	379 (46.3)	106 (28.0)	273 (72.0)	
3	80 (9.8)	40 (50.0)	40 (50.0)	
**Nodal status *n* (%)**				
0	501 (61.2)	130 (25.9)	371 (74.1)	*p* = .752
1	211 (25.8)	59 (28.0)	152 (72.0)	
2	93 (11.4)	28 (30.1)	65 (69.9)	
3	1 (0.1)	0 (0.0)	1 (100.0)	
**Overall Stage *n* (%)**				
1	267 (32.6)	55 (20.6)	212 (79.4)	***p = .001***
2	435 (53.2)	125 (28.7)	310 (71.3)	
3	116 (14.2)	44 (37.9)	72 (62.1)	
**Tumor grade *n* (%)**				
1	126 (15.4)	30 (23.8)	96 (76.2)	*p* = .171
2	272 (33.3)	62 (22.8)	210 (77.2)	
3	394 (48.2)	114 (28.9)	280 (71.1)	
**Presence of lymphovascular invasion *n* (%)**				
Yes	336 (43.4)	80 (39.6)	256 (44.7)	*p* = .211
No	439 (56.6)	122 (60.4)	317 (55.3)	
**Estrogen receptor *n* (%)**				
Positive	579 (70.8)	135 (23.3)	444 (76.7)	***p = .001***
Negative	230 (28.1)	81 (35.2)	149 (64.8)	
**Progesterone receptor *n* (%)**				
Positive	494 (60.4)	110 (22.3)	384 (77.7)	***p < .001***
Negative	315 (38.5)	106 (33.7)	209 (66.3)	
**Her2 receptor *n* (%)**				
Positive	203 (24.8)	28 (13.8)	175 (86.2)	***p < .001***
Negative	562 (68.7)	179 (31.9)	383 (68.1)	
**Menopausal status *n* (%)**				
Pre‐menopausal	431 (53.9)	119 (54.1)	268 (46.2)	*p* = .940
Post‐menopausal	369 (46.1)	101 (45.9)	312 (53.8)	
**Surgical type *n* (%)**				
Conservation	170 (26.4)	49 (22.0)	121 (20.4)	*p* = .630
Mastectomy	645 (73.6)	174 (78.0)	471 (79.6)	

The bold and italic values will refer to significant *p* values (ie. *p* < 0.05).

82.9% had presented with a clinically palpable breast lump. 13.1% were screen detected and hence, asymptomatic (Table S1). Others had nipple discharge, breast pain or skin changes and were subsequently found to have cancer on further evaluation. A higher proportion of asymptomatic women with IDC‐DCIS were found to have suspicious mammographic findings on screening which had included microcalcifications as compared to the group with No‐DCIS. This result was not found to be significant (*p* = .110) (Table S2). Nine patients presented with bilateral tumors, either synchronous or metachronous.

Lumpectomy was performed in 170 patients while 645 patients had mastectomy as a form of surgical treatment for their condition. One hundred and seventy four patients (78.0%) in the No‐DCIS group and 471 (79.6%) in the IDC‐DCIS group underwent mastectomy (*p* = .630).

Though majority of tumors in both groups were found to be grade 3 (48.2%), this result was not significant (*p* = .171). Similarly, there was no statistical significance upon comparison of the presence of lymphovascular invasion between both groups (*p* = .211).

The median size of the invasive tumor of the No‐DCIS group was 27 mm while the IDC‐DCIS group was 22 mm (*p* < .001). Patients with IDC‐DCIS were associated with lower T stage (*p* < .001) and better overall pathological stage (*p* = .001). Furthermore, patients with IDC‐DCIS were likely to express positivity in hormonal receptors (estrogen receptor, *p* = .001; progesterone receptor, *p* < .001). One hundred and seventy five patients (86.2%) with IDC‐DCIS were found to demonstrate Her2 positivity while 28 patients with No‐DCIS were found to be Her2 positive (*p* < .001). A total of 134 patients were found to have triple negative cancers. The mean duration of follow up was 93.7 months. Clinical outcomes have been summarized in Table 2. One hundred and fifteen distant recurrences were noted, with No‐DCIS group having 42 (20.5%) and the IDC‐DCIS group having 73 (13.6%) (*p* = .020). Forty eight local recurrences were recorded; No‐DCIS group had 17 (8.1%) while IDC‐DCIS group had 31 (5.8%) (*p* = .72).

**TABLE 2:** Recurrences of the entire study population and No‐DCIS and IDC‐DCIS study groups

Variable	Total (*n* = 818)	No‐DCIS (*n* = 224)	IDC‐DCIS (*n* = 594)	*p*‐value
**Local recurrence *n* (%)**				
Yes	48 (6.4)	17 (8.1)	31 (5.8)	*p* = .746
No	698 (93.6)	192 (91.9)	506 (94.2)	
**Distant recurrence *n* (%)**				
Yes	115 (15.5)	42 (20.5)	73 (13.6)	***p* = .020**
No	627 (84.5)	163 (79.5)	464 (86.4)	

The bold and italic values will refer to significant *p* values (ie. *p* < 0.05).

Adjuvant therapy received by both groups has been summarized in Table 3. The odds ratio of developing disease recurrence in the group No‐DCIS receiving hormonal therapy is 1.56 (95% CI: 1.05–2.32, *p* = .027). After adjusting for variables such as T and N stage, overall cancer stage, grade of tumor, presence of lymphovascular invasion, hormonal receptor status, this result was not significant (OR: 1.35, 95% CI 0.85–2.15, *p* = .205).

**TABLE 3:** Association between adjuvant therapy and risk of developing disease progression

Variable	Total (*n* = 818)	No‐DCIS (*n* = 224)	IDC‐DCIS (*n* = 594)	*p*‐value
**Hormonal therapy *n* (%)**				
Yes	533 (65.2)	128 (57.1)	405 (68.2)	***p* = .003**
No	270 (33.1)	92 (41.1)	179 (30.1)	
**Chemotherapy *n* (%)**				
Yes	462 (56.5)	128 (57.1)	334 (56.2)	*p* = .806
No	338 (41.3)	91 (40.6)	247 (41.6)	
**Radiation therapy *n* (%)**				
Yes	323 (39.5)	100 (44.6)	223 (37.5)	*p* = .051
No	474 (57.9)	117 (52.2)	357 (60.1)	

The bold and italic values will refer to significant *p* values (ie. *p* < 0.05).

Patients with No‐DCIS were 1.6 times more likely to develop disease progression in contrast to IDC‐DCIS (OR: 1.6, 95% CI: 1.1–2.6, *p* = .027) but this risk was not significant after adjusting for T and N stage, overall stage, grade of tumor, presence of lymphovascular invasion, hormonal receptor status (OR:1.4 95% CI: 0.9–2.2, *p* = .205). Patients with IDC‐DCIS had a longer mean of disease free progression as compared to those with No‐DCIS (13.2 vs. 12.5 years) (*p* = .014). The hazard ratio for disease free progression survival for the group with No‐DCIS was 1.6 (95% CI 1.1–2.2) (*p* = .014).

Patients with IDC‐DCIS recorded a longer mean survival than those with No‐DCIS (14.0 vs. 13.5 years, *p* = .095). Log rank test showed that this was not significant (*p* = .095). Multivariate analysis showed that both IDC‐DCIS and No‐DCIS have similar hazard after adjusting for variables such as age at diagnosis, T and N stage, overall stage, presence of lymphovascular invasion, type of surgery and hormonal therapy. (HR: 1.3 95% CI: 0.8–2.0, *p* = .307) (Table 4). The 5 year disease free survival for patients with IDC‐DCIS was 89.3% (95% CI 86.5%–91.9%) while the group with No‐DCIS was 84.5% (95% CI 78.9%–89.2%) (*p* = .071).

**TABLE 4:** Hazard ratio on survival analysis for IDC‐DCIS and No‐DCIS

	Univariate HR (95% CI)	*p*‐value
Group		
IDC‐DICS	**Reference**	.097
No‐DCIS	**1.43 (0.94, 2.17)**	
**Age of diagnosis**	**1.02 (1.002, 1.03)**	**.022**
**Hormonal therapy**		
No	**Reference**	**.032**
Yes	**0.64 (0.43, 0.96)**	
**Chemo therapy**		
No	**Reference**	.248
Yes	**0.79 (0.53, 1.18)**	
**Radiotherapy (DXT)**		
No	**Reference**	.912
Yes	**1.02 (0.68, 1.53)**	
**Age group**		
<40		
40–49	**0.66 (0.33, 1.32)**	.237
50–59	**0.76 (0.38, 1.54)**	.440
60–69	**0.77 (0.36, 1.64)**	.497
≥70	**1.49 (0.74, 2.97)**	.264
**Ethnicity**		
Chinese	**Reference**	
Malay	**1.21 (0.72, 2.04)**	.475
Indian	**1.51 (0.69, 3.30)**	.296
Others	**0.57 (0.18, 1.81)**	.339
**T stage**		
1	**Reference**	
2	**2.67 (1.61, 4.43)**	**<.001**
3	**3.59 (1.86, 6.34)**	**<.001**
**N stage**		
0	**Reference**	
1	**2.39 (1.49, 3.83)**	**<.001**
2 + 3	**3.26 (1.94, 5.47)**	**<.001**
**Overall stage**		
1	**Reference**	
2	**1.86 (1.04, 3.31)**	**.035**
3	**3.81 (2.06, 7.07)**	**<.001**
**Tumor grade**		
1	**Reference**	
2	**1.49 (0.59, 3.72)**	.399
3	**3.55 (1.54, 8.18)**	**.003**
**Presence of lymphovascular invasion**		**.003**
No	**Reference**	
Yes	**1.89 (1.25, 2.86)**	
**Estrogen receptor**		.070
Negative	**Reference**	
Positive	**0.68 (0.45, 1.03)**	
**Progesterone receptor**		.092
Negative	**Reference**	
Positive	**0.71 (0.47, 1.06)**	
**Her2 receptor**		.231
Negative	**Reference**	
Positive	**0.74 (0.45, 1.21)**	
**Menopausal status**		
Premenopausal	**Reference**	
Postmenopausal	**1.38 (0.92, 2.06)**	.121
**Surgical type**		
Conservation	**Reference**	**.013**
Mastectomy	**2.22 (1.19, 4.17)**	

	Univariate HR (95%CI)	*p*‐value	Adjusted HR (95% CI)	*p*‐value
**IDC‐DCIS** **No‐DCIS**	**Reference** 1**.43 (0.94, 2.17)**	**0.097**	**Reference** 1**.27 (0.80, 2.01)**	0.307

The bold and italic values will refer to significant *p* values (ie. *p* < 0.05).

**FIGURE 2:**
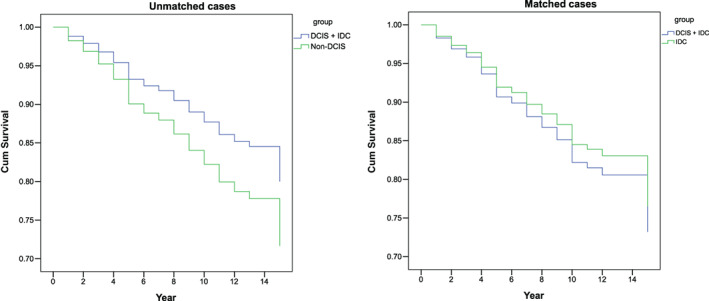
Breast cancer specific 5 year overall survival before (left) and after (right) propensity score matching

The breast cancer specific 5 year overall survival rate was performed in 163 pairs matched patients with the aid of propensity score matching (PSM) (Figure 2)and was 92.02% (95% CI 90.9%–94.7%). Cox proportion hazard ratio showed that patients with IDC‐DCIS more likely to progress faster to death (Table 5). Adjustment by age, overall tumor stage, menopausal status, hormonal receptor and cerB2 status showed no significant difference in the 5 year overall survival rate (*p* = .608).

**TABLE 5:** Utilization of Cox proportion hazard ratio

	Breast cancer related deaths (%)	Mean, years (95% CI)	HR (95% CI)	*p*‐value
IDC‐DCIS	25/163 (15.3)	13.30 (12.68, 13.92)	REF	
No‐DCIS	21/163 (12.9)	13.56 (12.96, 14.17)	0.86 (0.48, 1.54)	.608

*Note*: Adjustment for age, overall tumor stage, menopausal status, hormonal receptor, and cerB2 status.

The bold and italic values will refer to significant *p* values (ie. *p* < 0.05).

## 4 DISCUSSION

Existing biological studies are able to demonstrate a difference between mammary cancers with No‐DCIS and those with IDC‐DCIS. The current understanding is that these mammary tumors undergo cellular differentiation from the in situ form of disease into the invasive phenotype and this slow progression may suggest a possible favorable clinical prognosis.^7^, ^8^ To date, only a handful of Western studies were able to recognize this association where the presence of concomitant in situ disease possibly led to better prognostic features and clinical outcomes.^9^, ^10^ On the other hand, the role of DCIS in invasive mammary cancer and its clinical significance among the Asian population remains understudied.

In Singapore, screening mammography is highly subsidized, making it extremely affordable for asymptomatic women. With the increasing awareness for screening mammography, detection of early mammary cancers including the in situ tumors have also been on the rise. However, current treatment guideline for breast cancer is dependent on the pathological characteristics of the invasive component. Systemic treatment does not depend on the in situ component (DCIS) of the tumor.^11^

Microcalcifications, first detailed by Salomon in 1913, have been said to be one of the earliest mammographic feature that may suggest underlying early breast cancer including DCIS.^12^, ^13^, ^14^ Nearly 90% of women with DCIS do not present with palpable tumor, therefore suspicious appearing microcalcifications could be the only suggestion in such asymptomatic women.^15^, ^16^ Though our study results had suggested a higher proportion of asymptomatic women diagnosed with DCIS‐IDC to have abnormal mammographic findings as compared to the group with No‐DCIS, this finding was overall not significant. This could be because a large majority of our study subjects had presented with symptoms by the time mammography was performed. There has yet to be a common consensus between the clinical features and prognostic implications for tumors with concomitant DCIS and IDC. There has been increasing evidence to suggest that women with IDC‐DCIS may present at a younger age with smaller sized tumors with few or no nodal involvement.^9^, ^10^ Our results indicated that IDC‐DCIS is associated with smaller sized tumors and a lower overall clinical stage, thereby concurring with the hypothesis that tumors with concomitant DCIS were less biologically aggressive. Likewise, some studies have shown that tumors with concomitant DCIS are likely to express ER, PR, and cerB2 positivity as compared to tumors with No‐DCIS.^4^, ^17^, ^18^ Logullo et al had analyzed 155 sequential cases of T1cN0M0 ductal cancers, of which 51 had the component of DCIS. No correlation between DCIS and estrogen, progesterone receptors were found. While M. Dieterich did show tumors with IDC‐DCIS had better local recurrence survival rate as compared with those with pure IDC, there appeared to be no significant relationship between both groups of tumors and the hormonal markers that they expressed.^4^, ^5^ Our study results showed that IDC‐DCIS subjects were likely to express positivity in ER, PR, and cerB2 receptors and this was statistically significant. While our data suggest that IDC‐DCIS cancers may imply a less aggressive phenotype for patients with hormonal receptor or Her2 receptor positive cancers, we do acknowledge that triple negative malignancies may exhibit different biological behavior. Furthermore, Her2 receptor positive tumors appear to be heterogeneous and in many instances, may demonstrate equal aggressiveness as compared to the triple negative cancers.

Researchers have since supported the preliminary theory of this slow evolution in deriving the invasive component and thereby resulting in a possibly better clinical prognosis. This study did demonstrate that the group with No‐DCIS had higher percentage of distant recurrences compared to the group with IDC‐DCIS (20.6% vs. 13.5%, *p* = .020). However, we were unable to draw a similar conclusion for local recurrence. This might be attributed to the small total number of patients in our cohort who had developed local recurrences over the surveillance period as a result of better compliance to local radiation therapy prescribed. Furthermore, a large proportion of our study subjects had undergone mastectomy, which might in turn lead to lower local recurrences. Our study results also suggest that patients with No‐DCIS were 1.6 times more likely to develop disease progression as compared to those with IDC‐DCIS, in spite of the adjuvant treatment given. These results add weight to the current speculation that the absence of coexisting DCIS is associated with poorer prognostic features and outcomes among Asian patients, especially in terms of distant recurrences and disease progression. Unfortunately, due to our small sample size of breast cancer related deaths, we were unable to detect a significant difference in the breast cancer specific 5 year overall survival between the two groups.

The breast cancer specific 5 year overall survival rate was analyzed with PSM. No significant results were found between the group with No‐DCIS and IDC‐DCIS (92.02%, 95% CI = 90.9%–94.7%, *p* = .608). PSM is regarded as an advanced statistical technique to minimize any possible confounders in an observational study. It serves to reduce possible treatment assignment bias and mimic randomization.^19^ We utilized PSM to assess if concomitant DCIS affects the 5 year breast cancer overall survival rate after adjusting for certain covariates as mentioned above but we were unable demonstrate a more favorable breast cancer specific 5 year overall survival rate. It might be attributed to the fact that both genomic profiles are highly similar.^20^, ^21^

Lastly, we do recognize the limitations of this study. Being a retrospective analysis, any incomplete data namely, less detailed histology reports in the early years of the 21st century, had to be excluded. Other information such as patients' details may have been missing during the early days of data entry. Other inherent biases associated with retrospective study have to be considered. Secondly, analysis of tumor specimens had been performed in the absence of central pathologic review, hence establishment of details such as presence of isolated tumor cells (ITC) infiltration and micro metastases (mic) in lymph nodes were absent. Thirdly, our sample size was too small to demonstrate a significant difference in the 5 year breast cancer specific survival. Unlike the Western studies, the clinical outcomes among Asians such as recurrences and disease free outcomes as a result from tumors with coexisting DCIS has been understudied.^10^, ^22^ This study, with a long median follow up of 94 months, is to add weight to current findings and further strengthen the belief that coexisting DCIS does lead to better tumor profile. We are the first to document the clinical significance of concomitant DCIS in the presence of invasive cancers in terms of disease progression and distant recurrences.

## 5 CONCLUSION

With the limited data among Asian population, our study remains the first to demonstrate improved clinical outcomes in terms of disease progression and distant recurrences. Henceforth, this allows clinicians to better prognosticate and consider vigilant clinical surveillance of patients diagnosed with isolated IDC in remission.

## AUTHOR CONTRIBUTIONS

**Spoorthi Sudhakar Shetty:** Data curation (supporting); investigation (supporting); project administration (lead). **Chin Mui Jaime Seah:** Data curation (lead); resources (supporting). **Pei Ting Tan:** Formal analysis (lead); methodology (supporting). **Su Ming Tan:** Methodology (supporting); writing – review and editing (lead). **Wai Peng Lee:** Conceptualization (lead); data curation (supporting); writing – original draft (lead).

## Supporting information


**Table S1** Supporting information

## Data Availability

The data that support the findings of this study are available from the corresponding author upon reasonable request.
