# Supplementary material for: Does concomitant ductal carcinoma in situ affect the clinical outcome in breast cancer patients with invasive ductal carcinoma: An Asian perspective
Source: Cancer Rep (Hoboken). 2022 Jul 26;5(9):e1646. doi: 10.1002/cnr2.1646 (PMC9458486; doi:10.1002/cnr2.1646)
Supplement: Supplementary file 1 — Table S1 Supporting information [file CNR2-5-e1646-s001.doc]

| ***Variable*** | ***Total (n=818)*** | ***No-DCIS (n=224)*** | ***IDC -DCIS (n=594)*** |
| --- | --- | --- | --- |
| **Presenting complaint** |  |  |  |
| **Screening** |  |  |  |
| Mammographic | 68 (8.3) | 13 (5.8) | 55 (24.6) |
| Ultrasound | 18 (2.2) | 4 (1.8) | 14 (6.3) |
| Clinical | 21 (2.6) | 4 (1.8) | 17 (7.6) |
| **Symptomatic** |  |  |  |
| Lump | 678 (82.8) | 197 (87.9) | 481 (58.8) |
| Nipple discharge | 9 (1.1) | 0 (0) | 9 (1.5) |
| Nipple changes | 5 (0.6) | 1 (0.4) | 4 (0.7) |
| Pain | 15 (1.8) | 4 (1.8) | 11 (1.9) |
| Skin changes | 2 (0.2) | 0 (0) | 2 (0.3) |
| **Tumour laterality** |  |  |  |
| Left | 404 (49.4) | 108 (48.2) | 296 (49.8) |
| Right | 396 (48.4) | 108 (48.2) | 288 (48.5) |
| Bilateral | 9 (1.1) | 4 (1.8) | 5 (0.8) |
|  |  |  |  |

**Supplementary Table 1.**

| ***Variable*** | ***Total (n=818)*** | ***No-DCIS (n=224)*** | ***IDC -DCIS (n=594)*** | ***P value*** |
| --- | --- | --- | --- | --- |
| **Presenting complaint** |  |  |  |  |
| Screening | 107 (13.1) | 21 (9.4) | 86 (14.5) | 0.055 |
| Symptomatic | 709 (86.9) | 202 (90.6) | 507 (85.5) |  |
| **Mammographic screen detected** |  |  |  | 0.110 |
| No | 750 (91.7) | 211 (94.2) | 539 (90.7) |  |
| Yes | 68 (8.3) | 13 (5.8) | 55 (9.3) |  |

**Supplementary Table 2.**
